# CkREV Enhances the Drought Resistance of *Caragana korshinskii* through Regulating the Expression of Auxin Synthetase Gene *CkYUC5*

**DOI:** 10.3390/ijms23115902

**Published:** 2022-05-24

**Authors:** Jia-Yang Li, Jie-Jie Ren, Tian-Xin Zhang, Jin-Hao Cui, Chun-Mei Gong

**Affiliations:** 1 College of Horticulture, Northwest A&F University, Xianyang 712100, China; jiayang@nwafu.edu.cn (J.-Y.L.); renjiejie@nwafu.edu.cn (J.-J.R.); 2 College of Life Sciences, Northwest A&F University, Xianyang 712100, China; tianxinzhang@nwafu.edu.cn (T.-X.Z.); jinhaocui@nwafu.edu.cn (J.-H.C.)

**Keywords:** *Caragana korshinskii*, drought, auxin synthesis, HD-ZIP III, stress resistance

## Abstract

As a common abiotic stress, drought severely impairs the growth, development, and even survival of plants. Here we report a transcription factor, *Caragana korshinskii* REVOLUTA(CkREV), which can bidirectionally regulate the expression of the critical enzyme gene *CkYUC5* in auxin synthesis according to external environment changes, so as to control the biosynthesis of auxin and further enhance the drought resistance of plants. Quantitative analysis reveals that the expression level of both *CkYUC5* and *AtYUC5* is down-regulated after *C. korshinskii* and *Arabidopsis thaliana* are exposed to drought. Functional verification of CkREV reveals that CkREV up-regulates the expression of *AtYUC5* in transgenic *A. thaliana* under common conditions, while down-regulating it under drought conditions. Meanwhile, the expression of *CkYUC5* is also down-regulated in *C. korshinskii* leaves instantaneously overexpressing CkREV. We apply a dual-luciferase reporter system to discover that CkREV can bind to the promoter of *CkYUC5* to regulate its expression, which is further proved by EMSA and Y1H esxperiments. Functional verification of CkREV in *C. korshinskii* and transgenic *A. thaliana* shows that CkREV can regulate the expression of *CkYUC5* and *AtYUC5* in a contrary way, maintaining the equilibrium of plants between growth and drought resisting. CkREV can positively regulate the expression of *CkYUC5* to promote auxin synthesis in favor of growth under normal development. However, CkREV can also respond to external signals and negatively regulate the expression of *CkYUC5*, which inhibits auxin synthesis in order to reduce growth rate, lower water demands, and eventually improve the drought resistance of plants.

## 1. Introduction

Auxin primarily originates from Greek, meaning growth [1]. It is irreplaceable during plant growth and development, which influences plant apical growth, axillary bud formation, floral organ development, and root development [2,3,4,5]. On the cell level, auxin is competent in changing the plasticity of plant cells, facilitating cells to differentiate and elongate. Besides, auxin can also compose a sophisticated regulatory network together with diverse kinds of plant hormones, collectively accommodating plants to their surroundings [6]. Auxin biosynthesis mainly depends on the tryptophan pathway consisting of four chief branches, each of which can synthesize indoleacetic acid (IAA) catalyzed by different enzymes [2,3,7]. So far, only the IpyA pathway has been clearly proved. It mainly includes two steps. First, tryptophan is reversibly transformed to IpyA through transamination catalyzed by TRYPTOPHAN AMINOTRANSFERASE OF ARABIDOPSIS (TAA). Then, IpyA is irreversibly transformed to IAA through oxidative decarboxylation by YUCCA (YUC), a rate-limiting enzyme [8,9]. TAA/YUC pathway has been functionally proved in various plants serving as the chief biosynthesis pathway for endogenous auxin [10,11,12,13,14]. YUC5, one protein from the YUC family, plays an important role in regulating auxin biosynthesis.

ABA, salicylic acid, and ethylene are involved in plants’ responses to drought stress, and so is auxin [15,16]. Under stress conditions, *WES1*, a gene encoding IAA–amido synthase, from the GH3 gene family is up-regulated to inactivate IAA by binding it to an amino acid, which lowers the level of endogenous auxin and activates the expression of stress-related genes *PR-1* and *CBF* [17]. Aux/IAA and ARF are two significant protein families mediating auxin response, which directly regulate the expression of auxin early response genes. 31 *OsIAA* genes and 25 *OsARF* genes have been identified in *Oryza sativa*, among which *OsIAA2* and *OsIAA20* are up-regulated under high salinity conditions. The expression level of *O. sativa TLD1* from the *GH3.13* gene family is inhibited in tissues above ground under normal conditions while it is remarkably induced under drought conditions. The activation of the *TLD1* gene leads to the reduction in IAA concentration and the change of plant shape in *O. sativa tld1*–*D* gain-of-function mutant, which decreases plant water loss and improves survival rate [18]. During seed germination, *ntm2*–*1* mutant possesses strong salt resistance. Salt stress can induce *NTM2* to specifically bind to the promoter of *IAA30* and activate its high expression. Nevertheless, the high expression of *IAA30* induced by NaCl disappears in the *ntm2-1* mutant, which attenuates the inhibition of auxin on seed germination [19]. Moreover, TCPs are able to activate the expression of auxin synthesis genes, for example, *YUC8*, by facilitating the transcription activity of *PIF4* under high-temperature stress [20].

HD-ZIP III family significantly regulates the pattern formation of embryo, root, stem, and vascular bundle together with leaf development [21,22,23]. It was first reported in 1995 that *ATHB 8*, a member of the HD-ZIP III family in *A. thaliana*, could express after it was induced by auxin [24]. Further research found that auxin flow induced the expression of MONOPTEROS (MP), the latter would induce the expression of *PIN-FORMED 1* (*PIN1*), and polarly localized *PIN1* at a high expression level would promote the polarity flow of auxin. MP directly binds to the promoter of *ATHB8* to induce its expression while inducing *PIN1*. Interestingly, up-regulated *AtHB8* reduces the sensitivity of MP–induced *PIN1* to auxin, thus limiting auxin flow to a narrow scope [25] and stimulating precursor cells of procambium to differentiate into xylem cells at designated regions. The expression of other HD-ZIP III members, such as *ATHB15*, *PHV*, *PHB*, and *REV/IFL*, is down-regulated in *mp* mutants, which can be considered to be regulated by MP [26]. Besides, auxin biosynthesis genes *TAR1*, *TAR2*, and *YUCCA3*, *5*, *7*, *8*, *9* are indispensable for high expression of HD-ZIP III in primary root and the formation of metaxylem in *A. thaliana* [27]. Meanwhile, the HD-ZIP III family shares the same expression pattern with auxin [28,29,30]. Hence, the achievement of the function of the HD-ZIP family possesses a close relationship with auxin.

Drought is one of the major abiotic stresses. It triggers water deficiency that poses a severe threat to plants’ survival and yield [31]. *C.korshinskii* is widely distributed in relatively harsh environments. It has a variety of stress tolerance characteristics through adaptive evolution and plays an extremely important role in protecting the ecological environment and completing the ecological restoration [32]. It is an ideal material for studying the formation mechanism of plant adversity adaptation. Previous studies on *C. korshinskii* mainly focused on morphological anatomy, physiological ecology, etc., and its molecular regulation mechanism in response to abiotic stress needs to be further studied. Research focusing on the relationship between auxin and plant response to stress has been increasingly emphasized in recent years. Studies concentrating on the relationship between drought response genes and plant hormones in *A. thaliana* unveil that although the ABA-dependent pathway dominates plant response to drought stress, other plant hormones including auxin also have an impact on the expression of genes related to drought resistance [33]. Abundant studies have indicated that the synthesis and critical response genes of auxin are regulated by environmental stress, however, the response to stress of auxin and the regulatory mechanism thereof at the molecular level still requires elucidation. This paper reports a transcription factor called CkREV, a member of the HD-ZIP III family which can bidirectionally regulate the expression of the critical enzyme CkYUC5 in auxin synthesis according to external environment changes in *C. korshinskii*, a drought-resisting pioneer plant widely spread among the desert area in northwest China, and its mechanism of maintaining the equilibrium of plants between growth and drought resisting by controlling auxin biosynthesis.

## 2. Results

### 2.1. HD-ZIP III TFs Phylogenetic Analysis

**Figure 1:**
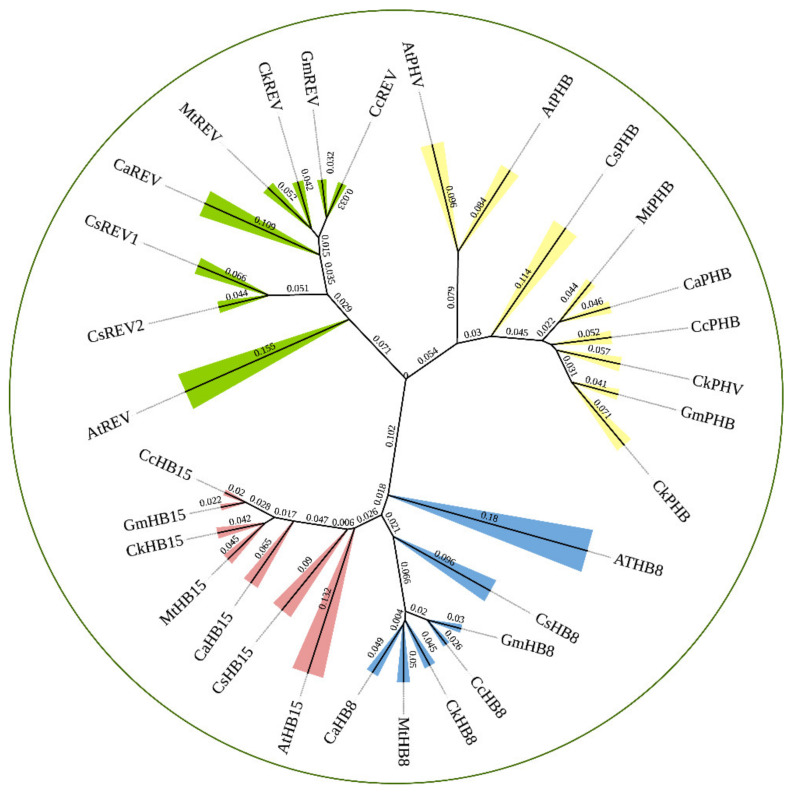
Phylogenetic analysis of HD-ZIP III family between *C. korshinskii, G. max, C. arietinum, M. truncatula, C. cajan, C. sinensis*, and *A. thaliana.* Bootstrap support (1000 repetitions) is shown for each node.

**Figure 2:**
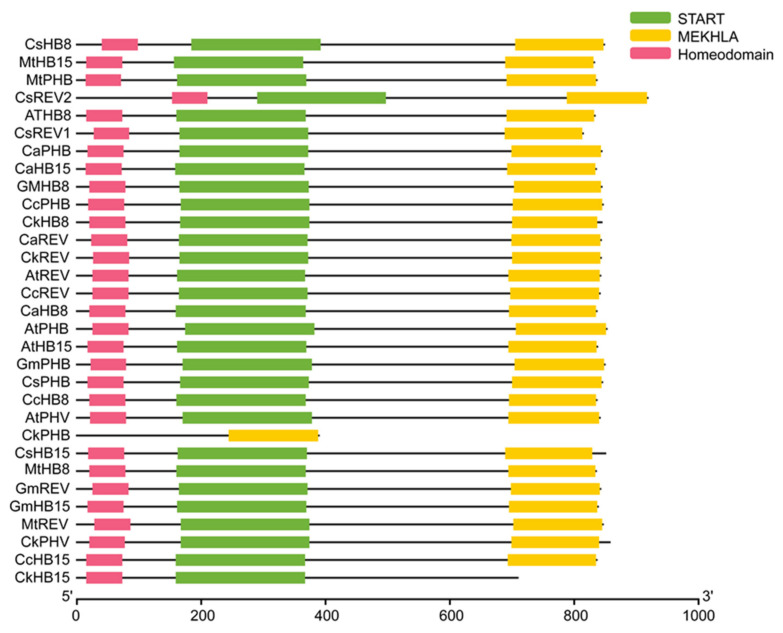
Conserved domain analysis of HD-ZIP III family between *C. korshinskii*, *G. max*, *C. arietinum*, *M. truncatula*, *C. cajan*, *C. sinensis*, and *A. thaliana*.

**Figure 3:**
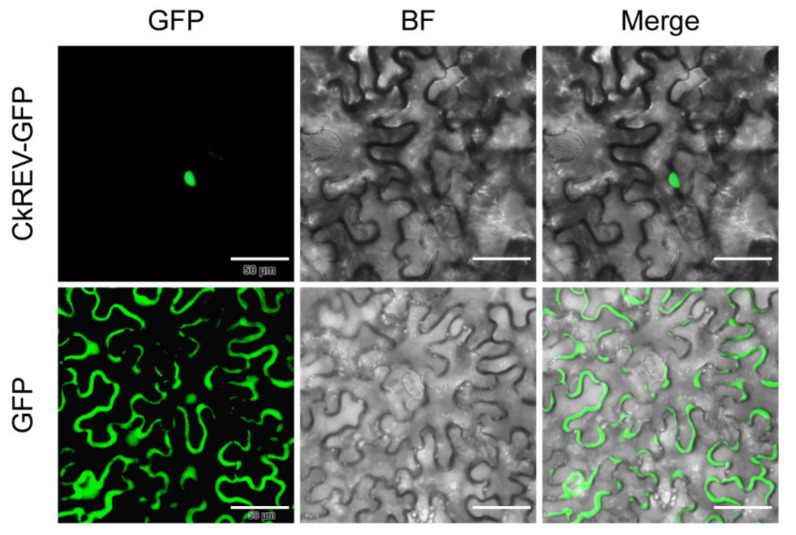
CkREV subcellular localization observation. Scale bars, 50 μm.

We constructed a phylogenetic tree including transcription factors of the HD-ZIP III family in *C. korshinskii*, *Glycine max*, *Cicer arietinum*, *Medicago truncatula*, *Cajanus cajan*, *Camellia sinensis*, and *A. thaliana* (Figure 1). 31 HD-ZIP III proteins derived from different species were categorized into four branches, among which the members of the HD-ZIP III family in *C. korshinskii* exhibited a relatively close relationship with those in *G. max* and *M. truncatula*, located in the same branch. On the contrary, REV exhibited a relatively distant relationship with other members of the HD-ZIP III family, implying that it probably possessed unknown regulatory functions different from others. We analyzed the conserved domain of the transcription factors of the HD-ZIP III family members of the above species through the pfam (http://pfam.xfam.org/) (accessed on 19 August 2021). The HD-ZIP III family of *C. korshinskii* including CkREV, has basically the same number and distribution of motifs as other species (Figure 2), and CkREV subcellular localization is also in the nucleus (Figure 3), which indicates that they may have similar biological functions.

### 2.2. CkREV Balances Plant Growth and Stress Resistance by Regulating the Expression of CkYUC5

**Figure 4:**
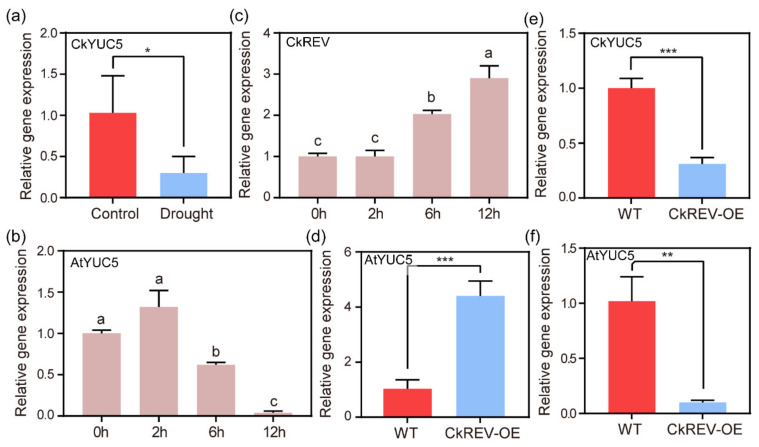
qRT–PCR analysis on the expression level of relevant genes. (**a**) Analysis of the expression level of *CkYUC5* in *C. korshinskii* leaves after natural drought treatment; (**b**) Analysis of the expression level of *AtYUC5* in *A. thaliana* seedlings after cultured on 1/2 MS medium for 7 days and transferred to PEG medium for simulated drought treatment; (**c**) Analysis of the expression level of *CkREV* in hydroponic *C. korshinskii* after drought simulation on PEG medium; (**d**) Analysis of the expression level of *AtYUC5* in *A. thaliana CkREV*–OE lines at the age of 4 weeks; (**e**) Analysis of the expression level of *CkYUC5* in *C. korshinskii* leaves instantaneously overexpressing CkREV; (**f**) Analysis of the expression level of *AtYUC5* in wild type and transgenic *A. thaliana* after drought treatment. (**a**,**d**–**f**) Data are shown as the mean ± SD of three independent experiments. Student’s *t*–test is employed to measure statistical significance between two samples with confidence level at 0.95 (*, *p* < 0.05; **, *p* < 0.01; ***, *p* < 0.001). (**b,c**) Data are shown as the mean ± SD of three independent experiments. One–way ANOVA was performed for the statistical analysis, where different letters represent significant differences (*p* < 0.05).

Under stress conditions, *WES1*, a gene encoding IAA–amido synthase, from the GH3 family is up-regulated to inactivate IAA by binding it to an amino acid, which lowers the level of endogenous auxin [16]. In addition to scavenging the existing IAA, can plants enhance their stress resistance by reducing their biosynthesis under stress? First, we treated *C. korshinskii* with drought conditions in order to explore the expression of the critical enzyme gene *CkYUC5* in auxin synthesis. We performed qRT–PCR to detect the expression level of *CkYUC5* (Figure 4a), which suggested that *CkYUC5* was down-regulated under drought conditions and auxin synthesis may be inhibited. The same outcomes were proved in *A. thaliana*, as it dropped precipitously with increasingly severe drought treatment (Figure 4b). In *A. thaliana*, the HD-ZIP III family has the same expression pattern as auxin [27,29,30]. Auxin biosynthesis genes *TAR1*, *TAR2*, and *YUCCA3*, *5*, *7*, *8* and *9* are necessary for the high expression of the HD-ZIP III family and the formation of metaxylem in *A. thaliana* primary roots [26], nevertheless, a member of the HD-ZIP III family called REV can directly bind to the promoter region of *YUC5* [34] and *LAX2*, *3* to regulate the synthesis and transport of auxin, respectively [35]. These two processes are closely related to the HD-ZIP III family, but the crosstalk between them under stress conditions has not been reported. In this study, we discovered that the expression level of *CkREV*, a member of the HD-ZIP family, was continuously up-regulated with increased drought levels (Figure 4c). Does CkREV mediate the negative regulation of *CkYUC5* or not?

We overexpressed *CkREV* in *A. thaliana* and performed qRT–PCR to test the expression level of *AtYUC5* in transgenic *A. thaliana*, the outcome of which indicated that the expression level of *AtYUC5* in *CkREV*–OE lines was conspicuously up-regulated (Figure 4d). Meanwhile, we instantaneously transformed *CkREV* into *C. korshinskii* leaves through plasmolysis and deplasmolysis to test the expression level of endogenous auxin synthetase gene *CkYUC5* after CkREV was overexpressed. Intriguingly, the expression level of *CkYUC5* is considerably down-regulated by CkREV (Figure 4e). The method we used to transform CkREV into *C. korshinskii* leaves caused osmotic stress similar to drought stress, which brought about the question of whether the contrary regulation mechanisms in CkREV stably transformed *A. thaliana* and instantaneously transformed the original plant were the consequences of osmotic stress during the transformation process. We made further efforts to raise wild type and CkREV–transformed *A. thaliana* under drought conditions and perceived that the expression of *AtYUC5* was remarkably down-regulated by CkREV under drought response (Figure 4f) while it was exceedingly up-regulated in normally cultured *A. thaliana CkREV*–OE lines.

### 2.3. CkREV Affects Auxin Biosynthesis by Regulating CkYUC5 and Inhibiting A. thaliana Root Length under Stress

**Figure 5:**
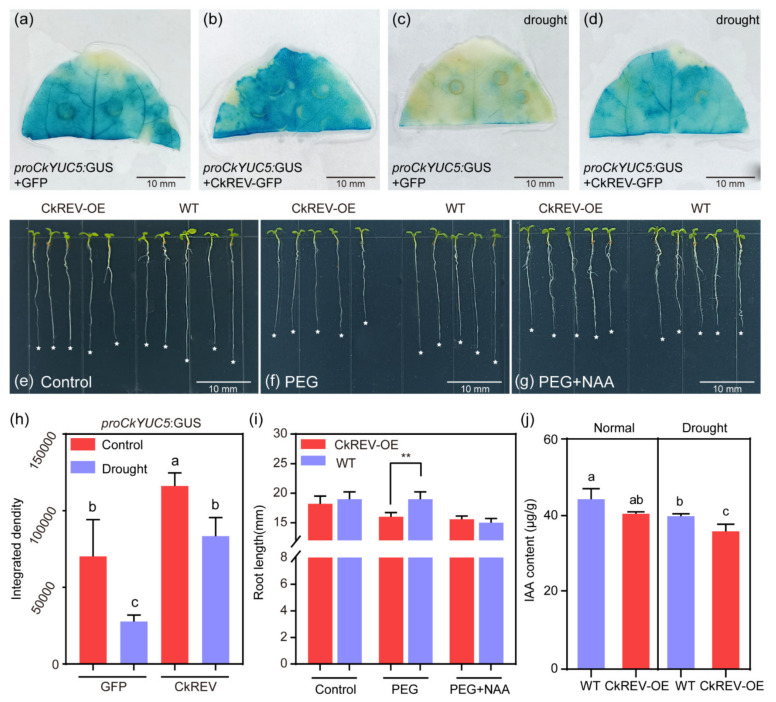
CkREV bidirectionally regulates the expression of *CkYUC5* and inhibits the *A. thaliana* root length under drought stress. (**a**–**d**) Observe the expression changes of *CkYUC5* under different treatment conditions through tobacco leaves cultured for 28 days (n = 3 biologically independent samples); (**a**) Localization of *CkYUC5* in the tip of tobacco leaf under normal culture conditions; (**b**) Localization of *CkYUC5* in the tip of tobacco leaf after *CkREV* overexpression; (**c**) Localization of *CkYUC5* in the tip of tobacco leaf after PEG treatment; (**d**) Localization of *CkYUC5* in the tip of tobacco leaf after overexpression of *CkREV* treated with PEG; (**e**–**g**) Four days after germination on 1/2MS plates, the phenotype of root length change of *A. thaliana CkREV*–OE strain and wild type under normal culture conditions, PEG treatment and PEG treatment with NAA added for 3 days (n = 5 biologically independent samples); (**h**) GUS staining statistics of *A. thaliana CkREV*–OE strain and wild type under different treatments; (**i**) Root length statistics of *A. thaliana CkREV*–OE strain and wild type under different treatments; (**j**) IAA content determination. Scale bars in (**a**–**g**), 10 mm. (**i**) Data are shown as the mean ± SD of five independent experiments; Student’s *t*–test is employed to measure statistical significance between two samples with a confidence level of 0.95 (**, *p* < 0.01). (**h**,**j**) Data are shown as the mean ± SD of three independent experiments. One–way ANOVA was performed for the statistical analysis, where different letters represent significant differences (*p* < 0.05).

The promoter of *CkYUC5* was constructed using the *pCambia1305* vector, and the expression pattern of *CkYUC5* gene was observed by the expression of the β–glucuronidase (GUS) gene. Under normal culture conditions, *CkYUC5* was abundantly expressed in the tip of tobacco leaves, which could be further up-regulated by the overexpression of *CkREV*. Consistent with the previous qRT–PCR results, the accumulation of GUS signals guided by the *CkYUC5* promoter was significantly inhibited after PEG treatment (Figure 5a–d,h). The root length of transgenic *A. thaliana* after PEG treatment was further analyzed. Under normal culture conditions, the *A. thaliana CkREV*–OE line showed no significant difference in root length compared with the wild–type, but after PEG treatment, the root length of *A. thaliana CkREV*–OE line was inhibited (Figure 5e,f). In *A. thaliana*, the balance between cell division and differentiation depends on the mutual regulation of the hormone cytokinin and auxin [36]. A previous study found that the free IAA content in the roots of *A. thaliana yucQ* mutants decreased by 55% compared to the wild type, and the lack of auxin significantly inhibited root elongation [37]. In order to further verify whether the inhibition of root length of the *A. thaliana CkREV*–OE line after PEG treatment was related to auxin deficiency, the transgenic *A. thaliana* was treated with PEG while adding 0.05 mg/L NAA, and the root length of *A. thaliana CkREV*–OE line returned to the level of wild type (Figure 5g,i). At the same time, the auxin content was detected in the *A. thaliana* seedlings treated as above. Not exactly as expected, under normal culture conditions, the IAA content in the *A. thaliana CkREV*–OE line did not show a significant difference from WT, but after drought stress treatment, the IAA content in the *CkREV*–OE line was decreased and significantly lower than WT (Figure 5j and Figure S1). In summary, CkREV affects the biosynthesis of auxin on the expression of *CkYUC5* in a different environment and plays an important role in regulating plant growth and stress adaptation.

### 2.4. CkREV Interacts with the Promoter of CkYUC5 to Regulate Its Expression

**Figure 6:**
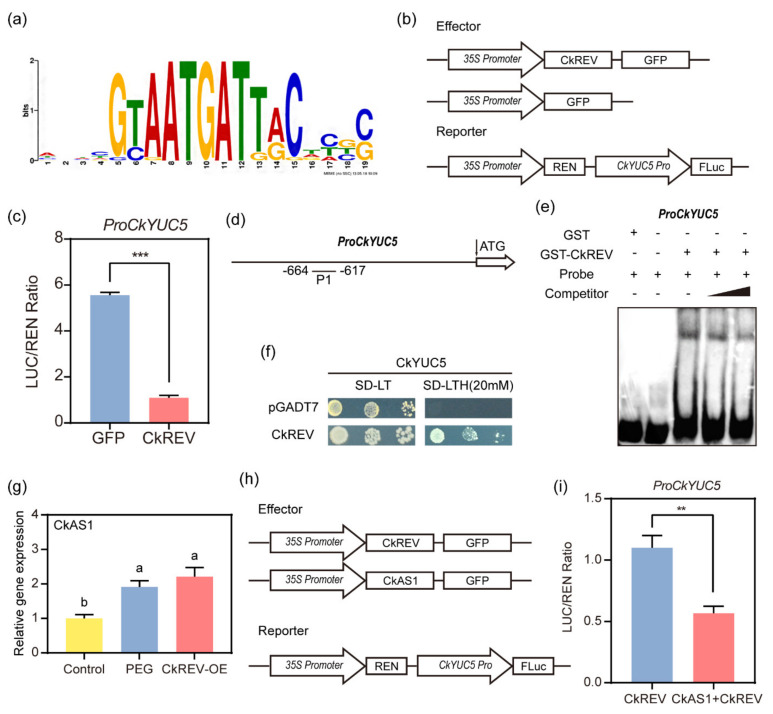
Interaction proof between CkREV and *CkYUC5*. (**a**) Recognition site of downstream genes of REV; (**b**) Dual–LUC experimental mode diagram; (**c**) Results of Dual–LUC experiment CkREV interacted with the promoter region of *CkYUC5* and negatively regulated its expression compared with GFP control group; (**d**) Sketch map of probe binding site in EMSA experiment; (**e**) Results of EMSA experiment indicated that CkREV–GST could directly bind to ATGAT element in the promoter region of critical enzyme gene *CkYUC5* in auxin synthesis; (**f**) The results of yeast one–hybrid further verified the binding of CkREV to the promoter region of *CkYUC5*; (**g**) qRT–PCR detection on *CkAS1* expression level in *C. korshinskii* leaves under different treatments; (**h**) Dual–LUC experimental mode diagram; (**i**) The Dual–LUC experiment was used to detect the effect of CkAS1 as an effector on the regulation of CkREV on *CkYUC5.* (**c**,**e**,**g**,**i**) Data are shown as the mean ± SD of three independent experiments. (**c**,**i**) Data are shown as the mean ± SD of three independent experiments. Student’s *t*–test is employed to measure statistical significance between two samples with a confidence level of 0.95 (**, *p* < 0.01; ***, *p* < 0.001). (**g**) Data are shown as the mean ± SD of three independent experiments. One–way ANOVA was performed for the statistical analysis, where different letters represent significant differences (*p* < 0.05).

The following critical question consists of whether CkREV directly or indirectly regulates *CkYUC5*. Since there was no public genome information about *C. korshinskii*, we firstly cloned the promoter of *CkYUC5* by genome walking and successfully obtained the unknown promoter sequence for 1144 bp in total upstream from *CkYUC5* after two rounds of nested PCR (Figure S2). We predicted transcription factor families probably regulating *CkYUC5* promoter region by PlantTFDB (http://planttfdb.gao-lab.org/) (accessed on 23 March 2021) and found 27 appropriate ones in aggregate, among which members of ERF and WRKY family extensively regulated that region (Table S1), in which a large number of light response elements were predicted by PlantCare as well (Figure S3). However, we also discovered that transcription factors from the HD-ZIP family potentially regulated it. We found that ATGAT is necessary for the binding of AtREV by searching its binding site on PLANT PAN (http://plantpan.itps.ncku.edu.tw/) (accessed on 26 March 2021) (Figure 6a). Being a homologous gene of AtREV, CkREV possesses a relatively conservative binding site. We constructed a Dual-luciferase reporter system with cloned *CkYUC5* promoter (Figure 6b) and injected it into Nicotiana tabacum to detect the activity of firefly luciferase and renilla luciferase. It turned out that CkREV could interact with *CkYUC5* and the former negatively regulated the expression of the latter remarkably (Figure 6c). We also discovered that the promoter region of *CkYUC5* possessed an ATGAT element, namely the binding site of CkREV. Based on that, we synthesized probes containing the core element and adjacent sequence (Figure 6d) with a biotin label linked to its 3′ end and cold probes without a biotin label for EMSA experiments in order to prove their interaction (Figure 6e). It turned out that CkREV could bind to the probes with a biotin label while cold probes competed with them. Therefore, it is proved that CkREV can bind to the ATGAT element in the promoter region of *CkYUC5* and regulate its expression. At the same time, we also used the yeast one-hybrid method for supplementary verification (Figure 6f), which further confirmed the regulation of CkREV on *CkYUC5*.

Interestingly, as a transcriptional activator, CkREV can negatively regulate the expression of *CkYUC5*. We speculate that there are other transcription factors involved in the regulation of CkREV. CkAS1, which is closely related to the function of CkREV, encodes a R2–R3 MYB domain protein that inhibits transcription. The results of qRT–PCR found that under PEG–simulated drought conditions CkAS1 in *C. korshinskii* leaves was significantly up-regulated, which is seen as CkREV was transiently overexpressed as well (Figure 6g). Therefore, CkAS1 may be involved in the negative regulation of CkREV on *CkYUC5*. Based on the previous Dual–LUC experiment, we injected two effectors CkAS1 and CkREV into tobacco and detected the expression of the reporter gene. Compared with CkREV alone CkREV+CkAS1 further down-regulated the expression of *CkYUC5* (Figure 6h,i).

### 2.5. CkREV Down–Regulates the Expression of YUC5 to Enhance the Drought-Resisting Ability of Plants under Drought Response

**Figure 7:**
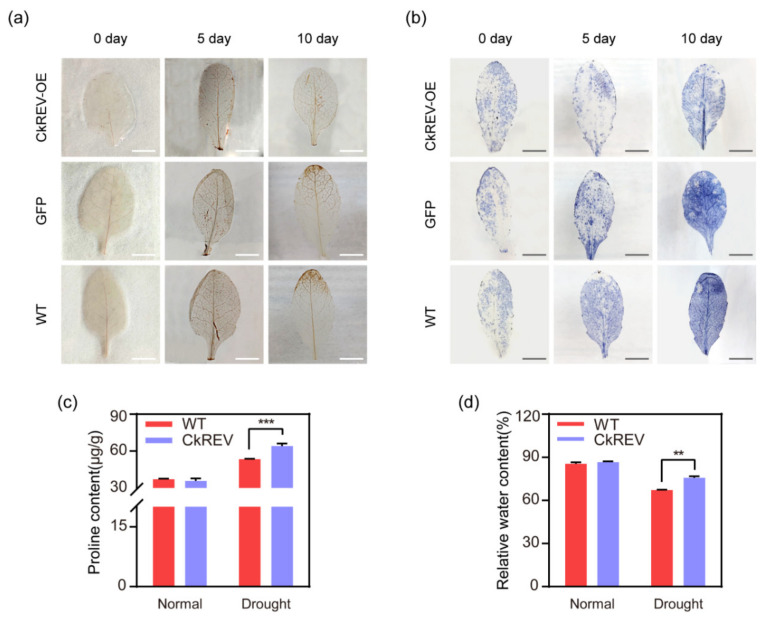
Phenotype analysis on transgenic *A. thaliana*. (**a**) DAB staining on rosette leaves at the same position after natural drought treatment for 0, 5, and 10 days (n = 3 biologically independent samples); (**b**) NBT staining on rosette leaves at the same position after natural drought treatment for 0, 5, and 10 days (n = 3 biologically independent samples); (**c**) Results of proline content determination; (**d**) Results of relative water content determination. (**c**,**d**) Data are shown as the mean ± SD of three independent experiments. Scale bars in (**a**,**b**), 5 mm. (**c**,**d**) Data are shown as the mean ± SD of three independent experiments. Student’s *t*–test is employed to measure statistical significance between two samples with a confidence level of 0.95 (**, *p* < 0.01; ***, *p* < 0.001).

Based on the results above, we discovered that CkREV would respond to external signals with improved expression levels when plants were confronted with drought stress. Its regulation on the expression of *CkYUC5* would transfer from positive to negative, which participates in the process of auxin reduction under stress conditions. So, what contributions do negative regulation of CkREV on auxin synthesis make to the adaptation of plants to drought stress? We treated transgenic *A. thaliana* with drought and detected the level of ROS in leaves by DAB (Figure 7a) and NBT (Figure 7b) staining. It turned out that *A. thaliana CkREV*–OE lines possessed ROS at a considerably lower level under drought stress. We also determined several drought resistance indexes for transgenic *A. thaliana*, such as proline content (Figure 7c) and relative water content (Figure 7d), the results of which indicated that *CkREV*–OE lines possessed rather exceptional traits for drought resistance, exhibiting excellent capability to adapt to drought stress. In summary, CkREV is conducive to the biosynthesis of auxin and the acceleration of plant growth by positively regulating the expression of the *CkYUC5* gene during normal development. However, when plants are subjected to drought stress, CkREV negatively regulates the expression of *CkYUC5* by sensing external signals and inhibits the biosynthesis of auxin, thereby slowing the growth rate of plants, reducing their demand for water, and enhancing the ability of plants to adapt to drought.

## 3. Discussion

The synthesis and metabolism, homeostasis regulation, polar transport, and signal transduction of auxin collectively influence its distributing gradient and how plants respond to it, which plays an indispensable role throughout the growth and development of plants. Auxin was primarily discovered to facilitate the growth of stems and roots [1]. Gradually, people perceived that it impacted a variety of physiological activities in plants, including senescence controlling, responses to abiotic stress and pathogens, formation of fruits, establishment and maintenance of cell polarity, apical dominance, phototropism, geotropism, etc. [4,5,38,39,40,41]. Auxin is synthesized in immature tissues such as tender leaves, cotyledons, and roots [42,43,44,45,46]. For roots, the synthesis of auxin aids in the maintenance of its concentration gradient, which is tremendously significant for normal growth and development [3].

**Figure 8:**
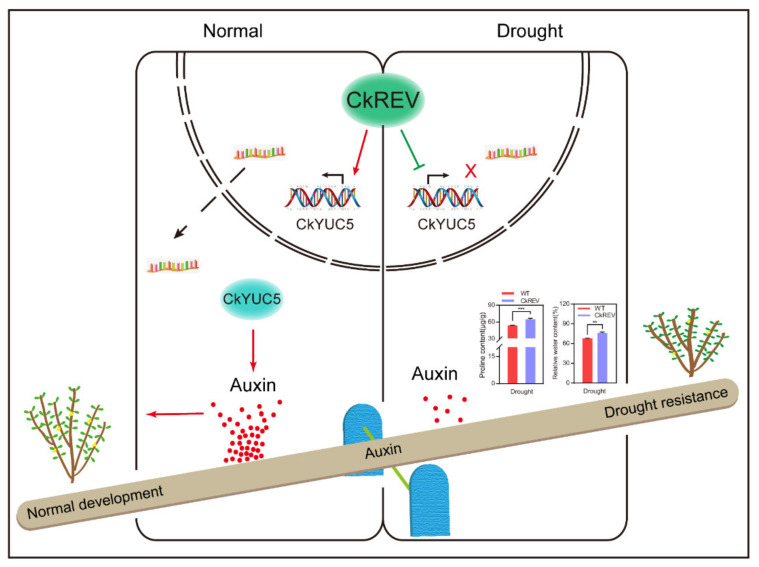
Model of regulation of CkREV on the expression of *CkYUC5*. The model concludes our research and exhibits that CkREV bidirectionally regulates the expression of *CkYUC5* critical in auxin synthesis depending on changes in external environmental signals, balancing the growth and drought-resistance of plants by influencing auxin synthesis.

Auxin also possesses a close relationship with stress. Recent research has indicated that growth retardation is the direct consequence of osmotic stress [47]. Abundant studies reveal that asymmetrical distribution of auxin is critical for plant development [48]. Overexpression of *ZmPIN1a* in *Zea mays* lowers the height of maize, increases the number of lateral roots, and inhibits their elongation, which assists in forming a well-developed root system and which improves its resistance to drought, lodging, and low phosphate environment [49]. Under stress conditions, the expression of the *WES1* gene encoding IAA-amido synthase from the GH3 family is up-regulated, deactivating IAA by catalyzing its binding to an amino acid, which enhances plant resistance through activating the expression of stress-related genes *PR-1* and *CBF* by lowering the level of endogenous auxin [17]. Direct determination of the content of endogenous IAA in leaves and roots indicates that plants living under salt stress and water deficiency conditions possess IAA at an apparently low level [50,51]. Further studies reveal that *O. Sativa* possesses seven *YUC* genes for auxin biosynthesis, six of which exhibit low expression levels under dry conditions [50]. The HD-ZIP III family shares the same expression pattern with auxin [28,29,30], the function of which is closely related to the synthesis and transport of auxin. In contrast to previous studies, we are surprised to find that CkREV, a member of the HD-ZIP III family, can bidirectionally regulate the expression of genes critical for auxin biosynthesis under normal and drought conditions (Figure 8). In the *A. thaliana CkREV*–OE line, the expression level of *AtYUC5* was significantly up-regulated, while after drought treatment, the expression level of *AtYUC5* shifted to be significantly down-regulated by CkREV (Figure 4d,f).

Studies focusing on the relationship between drought response genes in *A. thaliana* and plant hormones uncover that nearly 100 genes can respond to drought stress while responding to IAA [33]. The expression of *TLD1* from the GH3.13 gene family in *O. Sativa* is tremendously induced under drought stress. In *O. Sativa tld1*–*D* gain–of–function mutant, the activation of the *TLD1* gene leads to the decrease of IAA concentration and the change of plant shape, which abates water loss and improves survival rate [18].

Contrary to expectations, although the expression of *AtYUC5* was up-regulated, CkREV did not promote auxin accumulation under normal culture conditions. This might be related to the fact that the biosynthesis of auxin was tightly regulated in plants [52]. Conversely, drought treatment revealed that the IAA content in the *CkREV*–OE line was significantly lower than that in WT, while low levels of IAA facilitated plant resistance against drought stress [18]. The same pattern of regulation was also applied to *C. korshinskii CkYUC5* (Figure 5). *C. korshinskii* is a woody plant and is significantly different from *A. thaliana* in many aspects, including but not limited to responses toward biotic and abiotic stresses. In *Arabidopsis*, IAA can be synthesized by both tryptophan (Trp) -dependent and -independent ways, and the Trp–dependent way is much better characterized compared to the other one [14]. Although it is uncertain whether the remaining synthetic pathways are conserved in different species, as the major endogenous auxin biosynthesis pathway in plants, the conservation of the TAA/YUC pathway in the plant kingdom has been functionally checked in many plant species [10,11,12,13,14,53,54]. Therefore, under drought stress, CkREV downregulates auxin biosynthesis by negatively regulating the expression of *YUC5*, a key enzyme gene in the TAA/YUC pathway, and promotes plant adaptation under drought stress. This regulatory pattern may be conserved in many plant species.

Early in the 1970s, studies showed that plants with smaller cells possessed a stronger capability of resisting low water potential and water deficiency. Hence, rapid reaction to environmental changes and self-restriction on growth rate probably assist plants in surviving dry periods [55]. We utilized DAB and NBT staining to detect the level of ROS in *A. thaliana* leaves after drought treatment while determining relevant physiological indexes, the results of which indicated that *A. thaliana CkREV*–OE lines suffered less under drought conditions, exhibiting capability for drought adaptation (Figure 7).

## 4. Materials and Methods

### 4.1. Plant Materials

*C. korshinskii* samples employed originated from the experimental plot of Northwest A&F University, which was cultivated in soil after germination. Healthy and plump seeds were selected and washed with clean water. seeds were then wrapped up with wet gauze and left in a dark environment for germination for 3–5 days under room temperature before being transplanted into flowerpots.

*A. thaliana* samples employed were all Col-0 ecotype. The *p35S*:CkREV-GFP and *p35S*:GFP plasmids were transformed into *Agrobacterium tumefaciens* GV3101, which was used to infect *A. thaliana* Col-0 ecotype. Seeds were disinfected with 10% (*v*/*v*) sodium hypochlorite for 5 min and were sifted on 1/2 MS medium with hygromycin until homozygous T_3_ generation.

### 4.2. qRT–PCR Measurement

The total RNA of plant tissue was extracted by Plant RNA Isolation Kit (Beibei Bio, Zhengzhou, China). For each sample, we accurately absorbed 1 μg RNA according to RNA concentration of different treatments and repetitions. The first strand of cDNA was amplified by PrimeScriptTM RT reagent Kit with gDNA Eraser (Takara, Tokyo, Japan), and cDNA solution was diluted 3–5 times as the template of qRT-PCR. Quantitative analysis of the expression of relevant genes was performed on 2 × M5 HiPer Realtime PCR Super mix (Mei5bio, Beijing, China) and quantitative PCR Amplifier LightCycler 480 (Roche, Basel, Switzerland). Quantitative primers are listed in Table S2.

### 4.3. Genome Walking

We designed 3 specific R primers (SP1: TACCTAGCAGACTGAACACACTCGT, SP2: GAAGATGAAGCTTTAATCGGTCGTA, and SP3: TCTCTTAGGCGTACTGCTGTGGCTA), among which SP2 should be designed within SP1 and SP3 within SP2. There were no stringent requirements on the distance between every two primers, as 60–100 bp is preferable normally. Genome walking usually consists of nested PCR reactions for three rounds, each of which is thermal asymmetric interlaced PCR in two different annealing temperatures. A higher annealing temperature encourages the binding of specific primers while a lower one benefits that of universal primers. The sequence of universal primer and amplification program employed in genome walking complies with that in the literature cited [56].

### 4.4. GUS Staining

The *CkYUC5* promoter was amplified from *C. korshinskii* genomic DNA and inserted into *pCambia1305* vector. *p35s*::CkREV-GFP and *p35S*::GFP as effector plasmids were co-injected with *pCkYUC5*::GUS into tobacco leaves and cultured for 48 h. After different treatments, the GUS staining kit (Coolaber, Shanghai, China) was used to stain and observe the GUS expression level in the tip of the tobacco leaf.

### 4.5. IAA Content Detection

Four days after germination on 1/2 MS plates, the *A. thaliana* seedlings were further cultured for 3 days on plain MS medium and PEG–treated MS medium, respectively. Accurately weighing 0.1 g of *A. thaliana* seedlings before fully ground with liquid nitrogen, 0.9 mL of PBS (pH 7.4) solution was added to dissolve at a ratio of 1:9 (*w*/*v*), which was left at room temperature for 20 min to fully extract the IAA in the sample. Centrifuged at 3000 rpm for 20 min, the supernatant solution was the crude extract of plant IAA, and the IAA content in the plant was determined according to the instructions of the Plant Indole-3-acetic acid (IAA); Auxin ELISA Kit. (Jingmei, Yancheng, China).

### 4.6. Dual-LUC Assay

The promoters of target genes were amplified from the genome DNA of *C. korshinskii* and inserted into *pGreen II 0800-LUC* vector with *p35s*::*CkREV–GFP* and *p35s*::*GFP* used as effect plasmids. Reporter and effector were transformed into *Agrobacterium tumefaciens* GV3101 (pSoup-p19) and GV3101, respectively, which were cultured in a shaking incubator under constant temperature until the OD value was around 1.0. Bacteria were resuspended in solution with acetosyringone (AS) to OD 600 value was 0.8. Effector and reporter were combined in the proportion 8:2 and placed in a dark environment for activation for 2–4 h before being injected into leaves of *Nicotiana tabacum* at the age of 28 d. Samples were collected to test the activity of firefly luciferase and renilla luciferase by GloMax 20/20 Luminometer (Promega, Madison, USA) and Dual luciferase reporter assay system (Promega, Madison, WI, USA) reagents according to their instructions [57].

### 4.7. Electrophoretic Mobility Shift Assay (EMSA)

First, biotin label was linked to the 3′ end of artificially synthesized single-stranded oligonucleotide probe containing binding site by DNA 3′ end biotin label kit (Beyotime, Shanghai, China). Second, double-stranded DNA probe with biotin label was obtained by annealing with artificially synthesized complimentary chain. Third, purified CkREV protein was incubated with probe with biotin label at a certain proportion while unlabeled double-stranded probe was used as cold probe. Fourth, native-PAGE was employed to separate samples before being transferred onto nylon membrane (Solarbio, Beijing, China) with positive charge through wet transformation method. Fifth, the nylon membrane was placed under ultraviolet crosslinker purple (UVP, Upland, USA) at 254 nm, 120 mJ/cm^2^ for 60 s. Last, colour development was employed on a completely cross-linked nylon membrane by EMSA chemiluminescence kit (Beyotime, Shanghai, China) for observation under chemiluminescence imager. Detailed steps complied with instructions of EMSA chemiluminescence kit (Beyotime, Shanghai, China).

### 4.8. Yeast One–Hybrid

The transcription factor was cloned into the *pGADT7*–*rec2* vector, and the promoter fragment to be verified was cloned into the *pHIS2* vector. The above plasmids were co-transformed into yeast strain Y187 using the lithium acetate method, and screened by SD/–LT medium. The positive colonies that were successfully transformed were picked out in YPDA liquid medium, cultivated at 30 °C to OD 1.0, and diluted to 1:100 and 1:1000 three concentration gradients. We spotted the bacteria liquid on SD/–LTH plates containing 10-100 mM 3–AT for self-activation verification and performed experiments on the plate with 3–AT, the concentration of which inhibits self-activation.

### 4.9. DAB and NBT Staining

DAB can be oxidized by hydrogen peroxide into dark brown precipitates. Hence, DAB is employed as a dye to test the existence and distribution of hydrogen peroxide in plant cells. DAB solution was prepared at the concentration of 1 mg/mL and acidated by 0.2 M HCl to pH 3.0. 5 μL TWEEN–20 (0.05% *v*/*v*) and 0.5 mL 200 mM Na_2_HPO_4_ were added into the DAB solution while stirring, which produced DAB staining solution of 10 mM Na_2_HPO_4_ and increased pH again. Leaves were collected and absolutely immersed into DAB staining solution under vacuum and dark conditions for shake incubation for 4–5 h at the speed of 80–100 rpm, after which DAB solution was discarded and samples were bleached by solution (ethanol:acetic acid:glycerol = 3:1:1) before being photographed [58].

For NBT staining, leaves were immersed into 6 mM NBT solution prepared by citrate sodium buffer (pH 6.0) under vacuum and dark conditions for incubation for 5–8 h, after which NBT solution was discarded and samples were bleached by solution (ethanol:acetic acid:glycerol = 3:1:1) before being photographed [59].

## 5. Conclusions

The distinct expression patterns of *YUC5* under diverse environments explained that auxin biosynthesis in plants was stringently regulated. CkREV responded to external environment changes and further influenced the expression of *CkYUC5* and *AtYUC5* in contrary ways, indicating that the sensitivity of CkREV to the environment determines the regulatory directions of its downstream genes. Accordingly, CkREV can enhance the expression of *CkYUC5* in favor of plant growth during normal development while it can sense external signals to function conversely in order to decelerate plant growth and attenuate water demands confronted with drought stress. This research provides a novel pathway for expanding the nature of drought-resisting in *C. korshinskii*, offering choices of functional and regulatory genes for enhancing the drought-resistance of woody plants through biotechnology in desert areas from now on.

## Data Availability

The data presented in this study are available in the article or Supplementary Materials.

## Supplementary Materials

The following are available online at https://www.mdpi.com/article/10.3390/ijms23115902/s1.
