# Supplementary material for: CkREV Enhances the Drought Resistance of Caragana korshinskii through Regulating the Expression of Auxin Synthetase Gene CkYUC5
Source: Int J Mol Sci. 2022 May 24;23(11):5902. doi: 10.3390/ijms23115902 (PMC9180416; doi:10.3390/ijms23115902)
Supplement: Supplementary file 1 [file ijms-23-05902-s001.zip › Supplementary_Figures S1-S3.pdf]

CkREV enhances the drought resistance of *Caragana korshinskii* through regulating the expression of auxin synthetase gene *CkYUC5*

Jia-Yang Li [1], Jie-Jie Ren [1] , Tian-Xin Zhang [2], Jin-Hao Cui [2] and Chun-Mei Gong [1,*]

[1] College of Horticulture, Northwest A&F University, Yangling, Shaanxi Province,China; jiayang@nwafu.edu.cn(J.-Y.L.); renjiejie@nwafu.edu.cn(J.-J.R.)
[2] College of Life Sciences, Northwest A&F University, Yangling, Shaanxi Province,China; tianxinzhang@nwafu.edu.cn(T.-X.Z.); jinhaocui@nwafu.edu.cn (J.-H.C.)
* Correspondence: gcm228@nwafu.edu.cn(C.-M.G.)

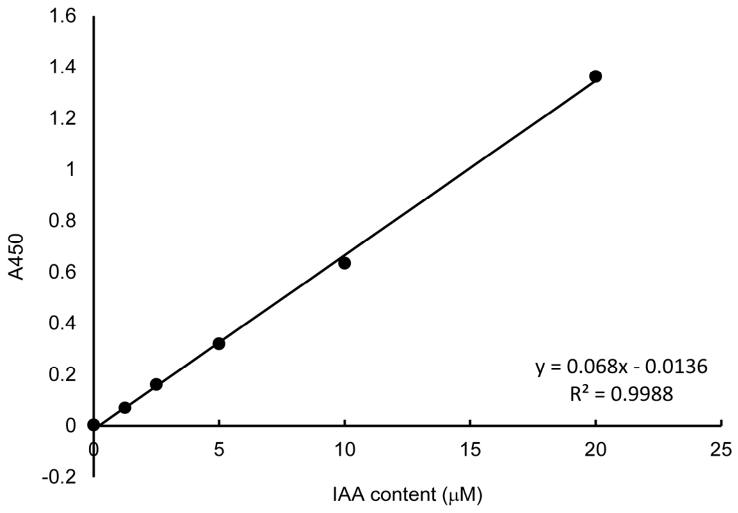

Figure S1. Standard curve of IAA content by ELISA.

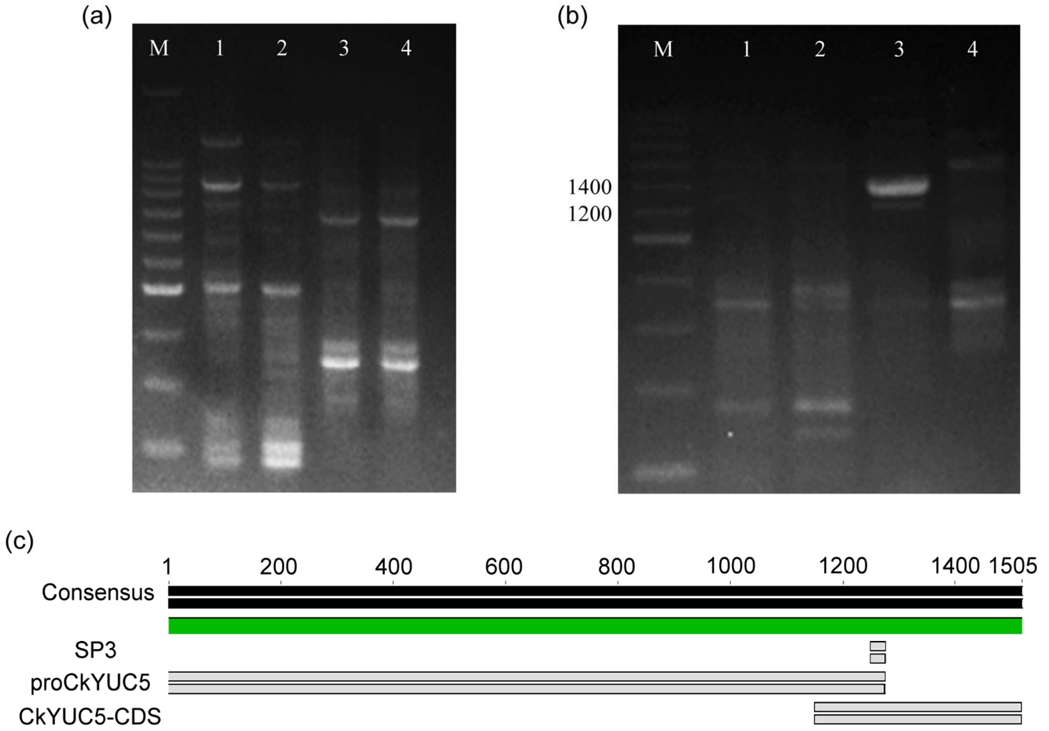

Figure S2. Genome walking clone *CkYUC5* 5' promoter sequence. (**a**) The first round amplification results of genome walking. M: marker. Lane 1-4: Different LAD primer combinations; (**b**) The second round amplification results of genome walking. M: marker. Lane 1-4: Different amplification templates and dilution multiples; (**c**) Sequence alignment results.

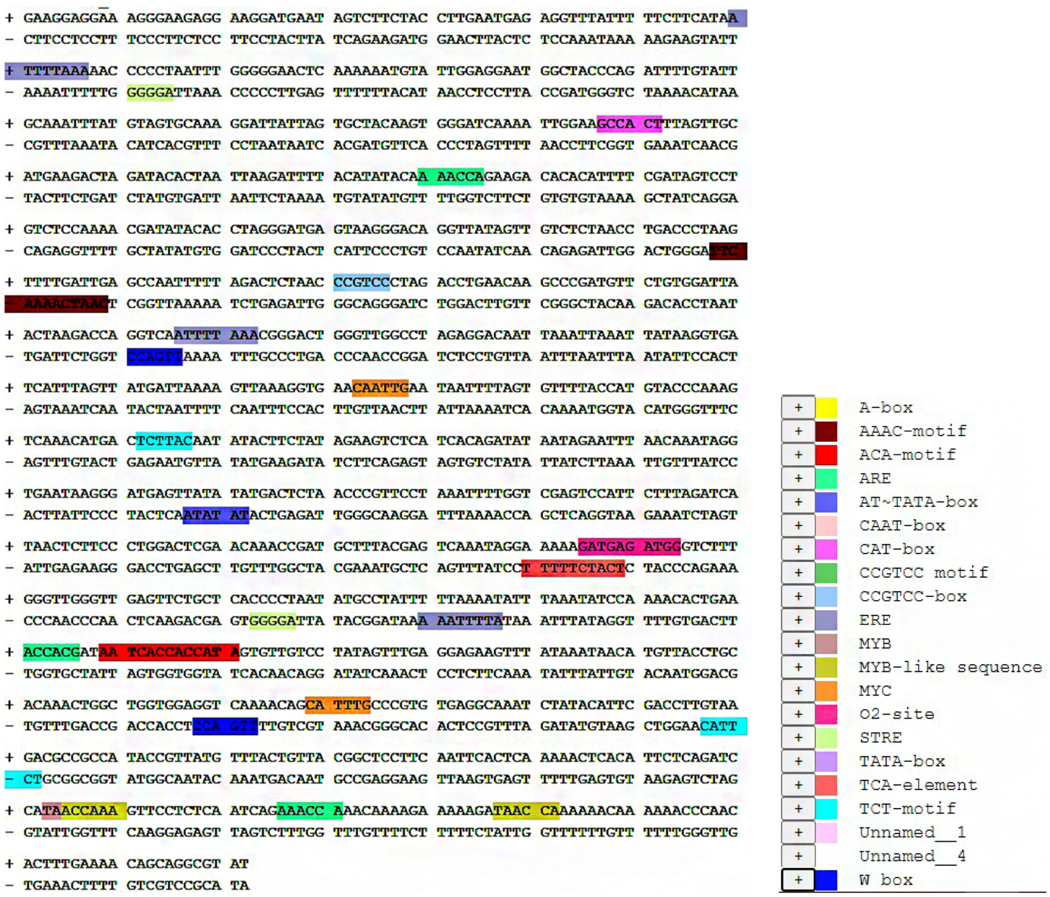

Figure S3. Cis-acting element prediction of *CkYUC5* promoter sequence. The Cis-acting element predicted using the PlantCare website (http://bioinformatics.psb.ugent.be/webtools/plantcare/html/).
