# Supplementary material for: CkREV Enhances the Drought Resistance of Caragana korshinskii through Regulating the Expression of Auxin Synthetase Gene CkYUC5
Source: Int J Mol Sci. 2022 May 24;23(11):5902. doi: 10.3390/ijms23115902 (PMC9180416; doi:10.3390/ijms23115902)
Supplement: Supplementary file 1 [file ijms-23-05902-s001.zip › Supplementary_tables S1-S2.pdf]

CkREV enhances the drought resistance of *Caragana korshinskii* through regulating the expression of auxin synthetase gene *CkYUC5*

Jia-Yang Li [1], Jie-Jie Ren [1] , Tian-Xin Zhang [2], Jin-Hao Cui [2] and Chun-Mei Gong [1,*]

[1] College of Horticulture, Northwest A&F University, Yangling, Shaanxi Province,China; jiayang@nwafu.edu.cn(J.-Y.L.); renjiejie@nwafu.edu.cn(J.-J.R.)

[2] College of Life Sciences, Northwest A&F University, Yangling, Shaanxi Province,China; tianxinzhang@nwafu.edu.cn(T.-X.Z.); jinhaocui@nwafu.edu.cn (J.-H.C.)

* Correspondence: gcm228@nwafu.edu.cn(C.-M.G.)

Table S1. The transcription factors bound by the promoter region of *CkYUC5* with plantTFDB prediction.

TF	Number	TF	Number	TF	Number	TF	Number
AP2	6	C2H2	17	LFY	2	TALE	5
ARF	2	CAMTA	1	MIKC_MADS	9	TCP	5
B3	2	Dof	9	MYB	14	Trihelix	1
BBR-BPC	5	ERF	87	MYB_related	4	WRKY	21
BES1	5	G2-like	1	NAC	2	YABBY	1
bHLH	6	GATA	4	Nin-like	1	ZF-HD	1
bZIP	3	LBD	7	SRS	5		

Table S2. qRT-PCR primer sequences of *C. korshinskii* and *A. thaliana.*

Primer name	Primer sequence(5’-3’)
Ckβ-actin-F	GGCTGTCCTCTCCCTCTATGC
Ckβ-actin-R	CGAACAATTTCCCGCTCAG
RT-*CkREV*-F	ATTCAAAACCAGGCGATAA
RT-*CkREV*-R	GAAATCACGCTGCGGACA
RT-*CkYUC5*-F	TTGAAAGGGCGGATTGC
RT-*CkYUC5*-R	CGTTGAACCGTGGGTAGAT
RT-*CkAS1*-F	GGTTAGGAAAGTGGTGGGAAG
RT-*CkAS1*-R	GCAATGCTGGTGCGGGG
Atβ-actin-F	TTACCCGATGGGCAAGTC
Atβ-actin-R	GCTCATACGGTCAGCGATAC
RT-*AtYUC5*-F	TTAGGCTCATGGGAAGTGAAGA
RT-*AtYUC5*-R	CGGGAAGGGCATTTTGG
